# “When they know that you are a sex worker, you will be the last person to be treated”: Perceptions and experiences of female sex workers in accessing HIV services in Uganda

**DOI:** 10.1186/s12914-017-0119-1

**Published:** 2017-05-05

**Authors:** Rhoda K. Wanyenze, Geofrey Musinguzi, Juliet Kiguli, Fred Nuwaha, Geoffrey Mujisha, Joshua Musinguzi, Jim Arinaitwe, Joseph K. B. Matovu

**Affiliations:** 1 Department of Disease Control and Environmental Health, Makerere University School of Public Health, Kampala, Uganda; 2 Department of Community Health & Behavioral Sciences, Makerere University School of Public Health, Kampala, Uganda; 3 MARPs Network, Kampala, Uganda; 4 Ministry of Health, Kampala, Uganda

**Keywords:** HIV/AIDS, Prevention, Care and treatment, Female sex workers, Uganda, Sub-Saharan Africa

## Abstract

**Background:**

HIV prevalence among female sex workers (FSWs) in high burden countries in sub-Saharan Africa varies between 24 and 72%, however their access to HIV services remains limited. This study explored FSWs’ perspectives of the barriers and opportunities to HIV service access in Uganda.

**Methods:**

The cross-sectional qualitative study was conducted between October and December 2013. Twenty-four focus group discussions were conducted with 190 FSWs in 12 districts. Data were analysed using manifest content analysis, using Atlas.ti software, based on the socio-ecological model.

**Results:**

FSWs indicated that HIV services were available and these included condoms, HIV testing and treatment, and management of sexually transmitted infections. However, access to HIV services was affected by several individual, societal, structural, and policy related barriers. Individual level factors included limited awareness of some prevention services, fears, and misconceptions while societal stigma was prominent. Structural and policy level barriers included inconvenient hours of operation of the clinics, inflexible facility based distribution of condoms, interuptions in the supply of condoms and other commodities, and limited package of services with virtually no access to lubricants, HIV pre- and post-exposure prophylaxis, and support following client perpetrated violence. Policies such as partner testing and involvement at antenatal care, and using only one facility for antiretroviral drug refills hindered HIV service uptake and retention in care. FSWs had major concerns with the quality of services especially discrimination and rude remarks from providers, denial or delay of services, and potential for breach of confidentiality. However, some FSWs reported positive experiences including interface with friendly providers and participated in formal and informal FSW groups, which supported them to access health services.

**Conclusion:**

Despite availability of services, FSWs faced major challenges in access to services. Comprehensive multilevel interventions targeting individual, societal, structural and policy level barriers are required to increase access to HIVservices among FSWs in Uganda. Policy and institutional adjustments should emphasize quality friendly services and expanding the package of services to meet the needs of FSWs.

## Background

Female sex workers (FSWs) world over, have a very high HIV prevalence and face major challenges in access to health services. This high burden of HIV and inequities in service access is more pronounced in the highest prevalence regions such as sub-Saharan Africa [1, 2]. Despite declining HIV prevalence and incidence in southern and eastern Africa, the 2016 Global AIDS update and prevention Gap Report show disproportionately high HIV prevalence among female sex workers. HIV prevalence among female sex workers in 10 countries with the highest burden ranges from 24% in Ethiopia to 72% in Lesotho [1, 2]. A cohort study among FSWs in Zimbabwe highlighted a high HIV prevalence (50%) and HIV seroconversion rate of 9.8/100 person years, yet only 41% of those who were HIV infected received antiretroviral therapy (ART) [3]. A national polling booth survey in Kenya showed that exposure to and utilization of HIV prevention services among key populations was least among FSWs [4].

The UNAIDS 2016 update estimates HIV prevalence among FSWs in Uganda at 34% [2]. In a systematic review of research among sex workers in Uganda, the HIV prevalence ranged from 32.4 to 52.0% (compared to 7.4% HIV prevalence in the general population), yet inconsistent condom use in the past month was prevalent (33.3–55.1%) [5]. According to the Modes of Transmission Study of 2014, HIV infections associated with FSWs accounted for 20.2% of new HIV infections in Uganda [6]. However, FSWs generally have poor access to HIV prevention and care services [7]. Further, HIV-related stigma, discrimination and punitive laws against commercial sex increase their vulnerability and may limit access to services [8].

Several models of service delivery among sex workers have been described including integrated facility-based and stand-alone targeted services as well as outreaches delivered at various hours through the day and night [9]. A survey among FSWs in four cities in South Africa, Mozambique, Kenya, and India showed variances in the choice of service providers across countries and the need to tailor service delivery models to the context [10]. However, interventions rarely integrate such contextual analysis or formal evaluations of the service outcomes and client satisfaction [1, 2, 9]. As such, FSWs continue to have limited access to services and those who make it into HIV care face challenges in sustaining care [11]. Vuylsteke B et al reported high attrition among sex workers in care in Ivory Coast, with only 55% retained at 24 months, and 47% at 36 months [12]. Several studies also report persistent barriers and especially poor quality services for sex workers [13–17].

In Uganda, the Ministry of Health (MOH) developed guidelines for service delivery among sex workers and many implementers have integrated these services [18]. It is however unclear to what extent these services meet the needs of the FSWs and what gaps still exist. In a qualitative study among sex workers in Harare, Zimbabwe, sex workers reported supply barriers including being demeaned and humiliated by health workers and social stigma surrounding their work. The demand barriers included competing time commitments, costs of transport and treatment, their marginalised status [17]. Shannon et al described structural and environmental factors external to individuals that interact and increase their vulnerability to HIV infection, including gender, cultural and economic inequities, and prohibitive government policies [19]. Yet, most studies have focused on individual level risks and few have explicitly assessed structural determinants of HIV and service uptake especially in areas with a high burden of HIV such as sub-Saharan Africa [20, 21]. This qualitative study explored multilevel barriers to HIV service access and opportunities for increasing access to services among FSWs in Uganda in order to inform and facilitate HIV service programming for these populations.

## Methods

### Study setting

This study was conducted among FSWs as part of a larger qualitative study among high risk groups including men who have sex with men [22, 23]. The study was conducted in 12 districts of Uganda including Kampala, Mukono, Rakai, Busia, Iganga, Mbale, Soroti, Lira, Gulu, Mbarara, Hoima and Bushenyi. Selection of the districts was based on geographical representation, HIV prevalence, and existence of “hot spots” along the transport corridors known for high concentration of mobile and high risk populations [24]. High concentrations of sex work and high HIV prevalence have been reported along the transport corridors and major towns in Uganda [8, 24].

### Study design and sample size

This was a cross-sectional study that employed qualitative methods of data collection, using focus group discussion (FGD) guides. Twenty-four (24) FGDs were conducted with 190 sex workers in 12 districts (i.e. 2 FGDs per district).

### Study tools

A FGD guide with open-ended questions was used to explore the FSW views on access to HIV services, their personal experiences and narratives, and opportunities for improvement. Answers to open-ended questions were followed by standard probes (verification, comparison and contrast) [22, 23]. The questions were constructed alongside the complex interplay between individual, networks, societal and policy related factors affecting access to HIV services among female sex workers [21].

### Sampling and data collection

The FGD participants were identified with the help of the known FSW-led organisations and non-governmental organisations (NGOs) that provide support to FSWs. FSWs from these groups, who were trained alongside the research assistants, identified and mobilized participants for the FGDs, ahead of the research assistants. From each district, an average of 16 sex workers was recruited to compose two FGDs. The FGDs were used to gather information on barriers to services and recommendations for service improvement, social networks and health seeking behaviors including access to services. The socio-demographic characteristics of participants including age, education, religion, marital status, and employment status were also collected.

### Quality control

To ensure quality, tools were pretested in communities outside the study districts and 1-week training was conducted to standardise procedures and ensure that all interviewers appreciate techniques for research among key populations, including sex workers. The training entailed a review of the study objectives, interviewing techniques with emphasis on special issues among sex workers, and detailed instructions on administering the interview guides. The training also addressed issues pertaining to how to gain entry into the communities and was facilitated by the investigators as well as selected members from the FSW community. Transcripts were reviewed by at least two investigators prior to entry into the Atlas.ti data managment software.

### Data management and analysis

Data were transcribed verbatim and translated into English. Each transcript was reviewed by at least two people who were fluent in both English and the local language. Data were organized with the help of Atlas.ti version 7, qualitative data management software. Data were analysed using manifest content analysis technique, based on theoretical constructs from the socio-ecological and modified socio-ecological models [21, 25, 26]. The socio-ecological model [SEM] posits that individual health behaviors and health outcomes are a result of complex interactions between individuals and the environment in which they live. This interaction takes place at various levels including at the intrapersonal, interpersonal; society, structural and policy levels. Using these levels, we examined the barriers that inhibit access to HIV services as well as opportunities to improve access to such services. The presentation of findings is organized around the SEM levels.

## Results

One hundred and ninety (190) FSWs were interviewed in 24 FGDs across 12 districts. The mean age of the participants was 25 years with a standard deviation of ±5.1. Majority of the participants (99; 53.8%) had primary or no education while 41.9% (77) had secondary level education. Sixty nine percent (126) had no other form of employment other than sex work. Most of the participants were Catholics (70; 38%) and Anglicans (57; 31%). The participants comprised largely women who had never married (85; 46.2%) and those who had divorced or separated (70; 38%). Of those who had children, most had one (54; 37.1%) or two children (49; 33.6%) (Table 1).

**Table 1:** Characteristics of the female sex workers who participated in the FGDs

Variable	*N* = 182^a^ (%)
Age (Years ± SD)	25 ± 5.1
18–20	39 (21.4)
21–25	71 (39.0)
> 25	72 (39.6)
Education	
None	10 (5.4)
Primary	89 (48.4)
S1–S4	64 (34.8)
S5–S6	13 (7.1)
Tertiary	8 (4.3)
Religion	
Catholic	70 (38)
Anglican	57 (31)
Muslim	47 (25.5)
Seventh Day Adventist	3 (1.6)
Pentecostal	7 (3.8)
Employment status	
Employed	58 (31.5)
Not employed	126 (68.5)
Marital status	
Never married/nor cohabited	85 (46.2)
Married/co-habiting	14 (7.6)
Divorced/separated	70 (38)
Widow	15 (8.2)
Parental status	
Have children	145 (78.8)
Have no children	39 (21.2)

^a^Eight of the 190 SWs had missing demographic data

### Access to services by sex workers

Participants reported that health services including condoms, HIV counseling and testing, diagnosis and treatment of sexually transmitted infections (STIs) and HIV treatment were available. Participants across all FGDs indicated that they were able to access these services although amidst difficulties due to several structural and policy related barriers.

### Barriers to services among sex workers

Sex workers narrated a number of barriers to seeking HIV prevention and treatment services, including individual level knowledge and awareness of services, misconceptions, fear of breach of confidentiality due to limited privacy at the health facilities, unwelcoming health workers and discrimination, as well as unfavourable hours of operation of health facilities and policies, as elaborated below.

### Intrapersonal factors

Although the environmental barriers were predominant, several intraperpersonal factors related to individual level knowledge, beliefs, and practices emerged.

#### Doubting the accuracy of HIV results

Respondents in several districts doubted their HIV test results, especially those who received HIV negative results, due to the belief that a sex worker cannot be HIV negative. Others reported having sex with clients who are known to be HIV positive and had difficulties accepting negative results.

“*What I am saying is that there are health workers who come here to test us in an outreach arrangement but those health workers lie to us because whoever they test, they say that she is [HIV] negative. I don’t understand it because whoever is tested and comes back says she is still negative*” (Busia FGD).

On the other hand, the fear to know their HIV status hindered some sex workers from accessing HIV testing services. They feared stigma but were especially concerned that this could affect their business.

#### Failure to adopt positive living

Participants noted that some of their colleagues are discouraged and fail to adopt positive living behaviors when they test HIV positive, especially if they are not counseled well.

“*Some of us are HIV positive. When some people know that they are HIV positive they tend to despise themselves and they don’t access drugs—all they care about is getting a man who will give them money, a drink and chicken…”* (FGD Soroti).  

#### Fear of harm

Although not often mentioned, some HIV-positive sex workers, in two districts, were hesitant to access HIV services due to the belief that the health workers may give them drugs that could kill them.

“*Sometimes when you go there regularly someone might get fed up of you and give you the medicines to kill you (n’akuwa kali akakujja mu nsi). My neighbor died about three months back. He was HIV positive and used to go to the facility every day. I think those doctors were tired of him and gave him a drug that killed him quickly”* (FGD Mbale).

#### Mobility of sex workers

Sex workers reported several challenges with receiving drugs and remaining on ART. They were often unable to continue treatment because they were always on the move in pursuit of clients. This was further complicated by inflexible clinic policies that did not allow them to get their refills at other facilities.

#### Inadequate knowledge about the availability of certain HIV prevention services

Sex workers across various groups noted the limited information about certain services especially support post-violence, lubricants, pre- and post-exposure prophylaxis. FSWs noted the high level of violelnce perpetrated by their clients including being raped and beaten if they did not consent to unprotected sex. However, they either feared to seek help or did not know where to get support. They expressed the need to use post-exposure prophylaxis but they did not know where and how to access it while a few noted challenges in accessing the services.

*“There’s normally that emergency treatment [PEP] in case a condom tears, or when they rape you but as sex workers we haven’t yet got that opportunity. I heard of someone who went for that treatment but the process was too long; going to the government hospital, then the police, I mean the process was too long ….”.* (Kampala venue based FGD).

*“A man can forcefully have unprotected sex with you, mind you it’s late in the night and you have nothing to do… If I have something to get me out of that danger like that emergency tab [PEP], it may help” (*FGD Busia).

Women in FGDs in Kampala were aware of the existence of lubricants and wanted to use them but had challenges accessing them. Very few respondents in FGDs ﻿outside Kampala knew about lubricants, however, they welcomed the idea when they were prompted, and noted that they purchased gels and related products on the market.

Although rarely mentioned, it was noted that some sex workers felt ashamed of being seen purchasing or asking for condoms from health facilities. Fear of being seen purchasing condoms was reportedly common among young sex workers and those in rural areas.

### Interpersonal factors: Social networks

Although some women feared HIV stigma within their social networks, the majority reported that the networks provided support to access HIV services. Sex workers in most of the districts had groups they belonged to, either at individual or community level. Most of the social groups were informal (unregistered with hardly any formal organizational structures). Majority of the social groups were mainly set up as revolving fund groups intended for financial empowerment. However, these groups also acted as “safety nets” for sex workers when they encountered problems such as conflict with clients, access to condoms, and family problems, among others. Women cited two FSW-groups in Kampala and one in each of the five upcountry districts (Soroti, Hoima, Gulu, Busia, and Lira); however, the rest of the districts had no ornanised groups. Some sex workers also reported that they never participated in any groups, even where they existed.

### Societal factors

#### Fear of stigma and discrimination by other sex workers, family and society

Sex workers hesitated to access services such as HIV testing and treatment due to fear of being stigmatized by fellow sex workers, their families and the community. They reported that being seen by fellow sex workers taking drugs associated with HIV would lead to someone being “*pointed fingers at*”. It was reported that some sex workers shun going for HIV testing due to fear of what their colleagues will think of them. FSWs noted that if they became HIV-positive while they are still engaged in sex work, their family members would not be willing to treat them or that they might chase them away from home. They also said that the community would blame them for getting HIV infection and would view it as a self-inflicted illness. 

### Structural and policy related factors

#### Privacy concerns and fear of breach of confidentiality

Respondents felt that the existence of healthcare facilities within the community did not necessarily translate into their utilization. Some FSWs were not confortable with facilities and providers who lived within their communities. They feared that these health workers might reveal information about their health status in the community. Privacy concerns were raised especially about public health facilities. FSWs feared to seek services such as HIV testing and treatment and to disclose to the health workers about their work. Some women felt that the health workers did not have the appropriate training to handle sex workers citing practices such as giving them their HIV results in non-private settings.

“*You know when you’re not HIV positive, they give you only one paper for your results but for one who is positive, they will always give you two papers. This makes everyone know that this person is positive; she has been given two papers with directions of where to find these people [HIV care providers]. But if these people were putting results on one side and directions on the other side of the same paper, it would be good because people would not know that one is positive*” (FGD Kampala).

#### Discriminatory and demeaning handling by health workers

Sex workers across all districts cited experiences of being denied treatment or delays to receive services due to their work.

“*When they know that you are a sex worker, you will be the last person to be treated or else they will tell you to come back the next day …*” (FGD Soroti).

In all districts, sex workers reported various forms of stigmatization at health facilities ranging from too many “*unnecessary*” questions about their sex lives to denying them the attention and care.

“*The other thing is too many questions these health workers ask; I don’t know if that’s the way they’re supposed to work but it’s too much. If you start asking me how many men I use then I don’t understand why, such questions even make you feel annoyed*” (FGD Kampala).

FSWs reported that the challenges they experience were most prominent at public and faith-based facilities. They noted that sex work is against the cultural and religious norms of society and at some faith-based facilities, FSWs were subjected to many unnecessary questions that make them uncomfortable.

*“… some of these health facilities are religious-affiliated like XX hospital is catholic [founded] so you tell a health worker that you’re a sex worker and it will be a crime because this thing is against the Bible, for a woman to use more than one man. This person will ask you a lot of questions, whether you’re happy about what you’re doing, etc not knowing that you don’t like that kind of life. Such health facilities don’t welcome us*” (FGD Kampala).

Because of these negative experiences and fears, several respondents in various FGDs said they would never reveal to any health worker about their work. Nevertheless, a few participants reported that some healthcare workers give them the necessarily care, acknowledge the challenges associated with being a sex worker and treat them well.

“*Health workers know that we face a lot of challenges as sex workers so when you tell them that you are one [a sex worker] s/he helps yo*u” (FGD Mbarara).

#### Unwelcoming behaviors exhibited by health workers towards sex workers

Health workers’ behaviors such as rudeness, discrimination and abuse, were cited as some of the unbecoming behaviors that make FSWs dread seeking HIV services at some health facilities.

“*I one time went to a health facility, I had a tummy problem and this nurse asked me whether I was a sex worker. When I agreed, she asked me why I went for that [sex work] saying that maybe I had contracted syphilis…we argued and I told her to work on me because I was going to pay for her services…*” (FGD Gulu)

Respondents also narrated negative body language and non-verbal signs of rejection by health workers.

“*… You can read the health worker’s face which shows that he is looking at something very bad and it makes you feel very bad. When they see us, they see us as animals.*” (FGD Kampala).

#### Delays and unfavorable working time at health facilities

Participants expressed dissatisfaction with the pace at which they were served in public facilities and the time when the health facilities were operational. Given the nature of their work which involves working at night, FSWs found it very inconvenient to spend the entire day at health facilities; many times going back home without receiving the services they wanted. For those on HIV treatment, delays in receiving services and missing required services could potentially affect their adherence and retention. The FSWs noted that those with money resort to private facilities where services are faster.

*“Now like us who’re HIV positive you can stay at the health facility for the whole day unless you go to those health facilities that understand us sex workers but not everyone can afford go to such health facilities. You spend the whole day at this health facility yet at night you have to work, which makes life hard”* (FGD Kampala).

### Costs involved in obtaining a service

Many of the participants noted that they were into the sex work only because of poverty. They felt the little money that they earned from sex work was not enough to meet their basic needs such as food, so, they found it hard to pay for health services. They highlighted expectations of tips by health care workers as an additional burden and the general lack of money for health care as a barrier to access to general health care and HIV services by sex workers.

“*Those who give the drugs want to be tipped before or at times the lines are too long that is the reason why some people cross to Kenya than getting them [health services] here in Uganda*…” (FGD Busia)

Some FSWs reported that even if the cost of getting services was low, they would still not afford it. For instance, several FSWs noted that they could not afford to use condoms unless they are availed to them free of charge. FSWs indicated that they usually find it difficult to get money for transport to the health facilities especially when they have to make repeat visits for services such as CD4 tests, condoms and HIV testing due to stock out of supplies. Those in rural areas also cited long distances to health facilities.

“*Imagine when you are working from the lake shore which is more than 50 miles from here. You board a vehicle to come and check your CD4 levels. That means you pay 20,000Shs to and 20,000 Shs back which comes to 40,000Shs. Imagine you fail to get your CD4 tested and all they tell you is to come the following week. You come the following week and the same thing happens. And sometimes you find that you don’t have money to bring you back, you know the life of a widow*” (FGD Hoima)

#### Challenges with access to condoms

Most sex workers accessed condoms within and outside their places of work. They accessed free condoms which were delivered to their lodges by various organizations through community outreach programs. Condoms were also available at different hot spots such as taxi stages and at health facilities. However, there were often interuptions in the condom supplies delivered through the community networks and FSW were forced to go to facilities for condoms. However, in some health facilities access to condoms was limited to only patients and condoms were rationed while other facilities also experienced stock outs.

*“When you go to the hospitals you will not be given the condoms unless you are sick and you have gone to get your drugs. You are not allowed to drop in and get condoms” (FGD Gulu).* 

The women in the venue-based FGD in Kampala felt they also needed lubricants but these were completely unavailable. 

### Unfavorable policies at the health facilities and other issues of concern

#### Partner involvement in HIV testing and PMTCT services

FSWs did not like the practice of being told to go to health facilities with their male partners for services, for example HIV testing and PMTCT services. They noted that they have many partners and fear disclosing to the health workers that they are sex workers. They also felt that their sexual partners may not want to be identified with them, and would thus be unwilling to go to the health facilities with them.

*“For me I have got a man who is wedded and he has made me pregnant and when I go to the hospital to get treatment or checkups the nurses always tell me to go and bring my man and yet the man does not want to expose himself as the father of the child because he is wedded” (FGD Soroti)*

*“The other thing is that I might be having a lover who doesn’t know that I am a sex worker so I can’t just get such a person and take him to that place where he will know what I am (FGD Lira).*

#### Unfavorable ARV dispensing policies

FSWs reported that the health facilities which they approached for drug refills refered them back to the clinics were they registred, which is not always feasible as they may not have transport and time to go back.

“….*as they tell me to start on drugs Katosi is not where I am going to stay I may want to go to Gulu because at times it is where I am based. Yet I may not have transport to bring me back to Katosi. It is hard to change a place to get drugs from because it involves asking and answering so many questions*” (FGD Mukono)

#### Legal barriers

Sex work is illegal in Uganda and several sex workers mentioned that they were frequently arrested. Some sex workers who had been previously arrested reported that they missed their treatment. Those who encountered violence hesistated to seek help for fear that they might be arrested.

### Opportunities for increasing access to services among sex workers

#### The role of social networks in HIV prevention

Participants noted that belonging to social networks had a positive influence on their adoption of HIV/STI prevention and treatment services. They mentioned that members in the network usually encourage each other to test for HIV and access treatment if found to be HIV positive. Women who were already on treatment counseled those who had recently started treatment and, where necessary, escorted the fearful ones to health facilities to access HIV testing and treatment. Sometimes, members raised money for others who did not have transport to facilities; encouraged each other to use condoms consistently; and/or supplied condoms to those who did not have them.

*“Usually when we get a new member we ask them to go and get tested because most of us have tested and we are positive so if we find that the new member is positive then we make sure she starts on medication”* (FGD Hoima).

In case a member becomes ill or is unable to get any client to make money to cater for her basic needs such as food, the group raises money for her. At times, network members collect medication for their colleagues who cannot make it to the health facilities.

“*We make sure that the old people [more experienced] on treatment help the new people starting treatment. For example, we can register an old person to collect and deliver the drugs for the new person*” (FGD Hoima).  

In addition, network members can rescue their colleagues when clients force them to have unprotected sex. The rescue involves ganging up and beating the client, if needed. Most women noted that the existing social groups for sex workers can be empowered to extend HIV services to sex workers, including distribution of condoms.

#### Use of media to promote HIV programs

The media can be a useful channel for promoting HIV programs. Indeed, in some FGDs, respondents noted that they were encouraged to test for HIV through television and radio messages and community loud speakers.

“*We’re encouraged by radios, TVs … presenters on radios such as Mega FM encourage us every Friday to test and know our status from any referral hospital where they offer such services*” (FGD Gulu).

#### Establish specific health facilities or clinics for sex workers

All participants across the different districts voiced a need for establishing specific facilities for sex workers where they can easily access HIV services without fear of being discriminated, embarrassed or treated in a demeaning way.

*“I would like them [HIV service providers] to provide us with a clinic here in Soroti for the sex workers, so that in case someone has got a problem they will have no problem going there. We would feel free to talk to the doctors because they know that we are sex workers and they know the kind of problems that we face”* (FGD Soroti). 

Participants suggested a need to identify a contact person for sex workers, preferably a peer, within the government health facilities to address FSW-specific issues and ensure that FSWs get the required services. Other suggestions included having a special desk for sex workers, a dedicated health worker to attend to them and specific days for FSW services. They also suggested the use of special cards for sex workers which they would present to the health facilities and be attended to very fast. 

#### Community distribution of ARVs, outreach services and moonlight clinics

Participants felt that services such as distribution of ARVs should be introduced within their communities to improve access since sex workers fail to go for drug refills at the health facilities due to lack of money for transport. Participants also proposed establishing several drug distribution outlets in the community.

“*They [health providers] should help us and make sure that in every town or trading centre, there are ARV outlets, so that we can collect the drugs from wherever we are. They could give us cards which identify us, which we could present to any clinic, and we get the drugs*” (FGD Iganga).

Most of those who had tested for HIV tested through outreach programs, which they felt were friendlier, called for increased support for such programs to reach out to all sex workers with HIV testing, condoms and other HIV services, including treatment. They also proposed night or “moonlight” clinics.

#### Provision of health information on phones

When asked if use of phones can improve access to health information among sex workers, some participants had reservations since phones are not universally available while others felt the messages would need to be translated into several languages. However, most of the sex workers felt that it would be a good idea to use phones to disseminate health information while maximizing confidentiality.

#### Motivation and sensitization of health workers to serve sex workers better

Participants suggested a need to sensitize health workers on the needs of sex workers; motivate them through salary increments or increase provider numbers to reduce delays at health facilities.

“*We would like these health workers to be sensitized on how to handle patients because by the time this patient comes to a health facility s/he is in pain but these female health workers are rude and even abuse you ………”,* (FGD Rakai).

To create fairness in service delivery, respondents suggested that strict penalties should be instituted for taking bribes.

#### Engagement of law enforcement agencies in HIV prevention programs

Some FGD participants highlighted the challenges faced in providing services to sex workers within the current context where sex work is illegal and suggested the need to engage and involve the law enforcement agencies.

## Discussion

This study explored the multi-level challenges faced by FSWs in accessing HIV services and opportunities for improvement. We found that HIV services were widespread across the districts. However, FSWs faced a lot of barriers in accessing these services. Individual level barriers included limited information about certain cervices, misconceptions leading to denial of HIV negative results, fear of HIV positive results and stigma. Societal factors included community stigma related to sex work and HIV positive status within the networks of sex workers, the general community and among providers. Many structural and policy related challenges emerged including stigma and discrimination by providers, concerns about confidentiality within facilities, unfriendly services due to the inconvenient operating hours of the clinics, limited access to some services such as PrEP, PEP, and support post-violence, and perceived inadquate provider skills to handle sex workers. Sex workers faced challenges in ensuring continuity of HIV treatment because of high mobility and inflexible clinic policies that restricted refills to only one facility while arrests by law enforcement officers interfered with their treatment. The requirement to present their male partners for HIV testing at antenatal services was also a challenge to the FSWs. General health care quality challenges (e.g. long distances to health facilities, long waiting time, and stock out of supplies) were also prominent in addition to social challenges such as poverty and client-perpetrated violence.

Whereas some of these barriers have been reported [13–17, 27], few studies have comprehensively assessed the multi-level barriers and opportunities for increasing access to HIV services among female sex workers within the same study, in high burden countries in sub-Saharan Africa. Studies in Ethiopia, Kenya, Uganda, Zimbabwe, and South Africa reported discriminatory behaviour from healthcare workers and a lack of dedicated services for sex workers [28–30]. Our findings highlight several multi-level challenges and gaps in exisiting national guidelines and service delivery that call for more comprehensive individual and community behavioral and structureal interventions [18]. Based on our findings, it is evident that further community level mobilization is necessary to address knowledge gaps and misconceptions. This should be coupled with provision of a comprehensive package of quality services that address the needs of FSWs. Notable concerns include irregular supply of condoms and supply through facilities, virtually no access to lubricants, PEP, and support after gender-based violence. Due to limited access to lubricants, some women were using lubricants that could affect the integrity of the condoms. This challenge was also prominent in our analysis of experiences among MSM [22, 23]. Additionally, sex workers raised concerns about the hours of operation for the clinics and the long distance to facilities. Distribution of condoms and lubricants through the sex worker networks and outreach services were suggested as potential options for addressing some of these limitations. Further, HIV treatment guidelines and implementation should enhance access to and continuity of treatment for mobile populations such as FSWs, by integrating mechanisms for refills at multiple sites. While emphasizing male partner testing and access to services, providers should be sensitive to the circumstances of FSW and other populations that may not be able to present such partners. To expand the reach of services for FSWs, it is necessary to offer more friendly services in the more widespread network of public facilities rather than focus on only CSOs. A systematic review of intervention packages and service-delivery models, and extent of government involvement in FSW services in Africa revealed that most projects were small and research-based, were not well coordinated, had minimal government involvement and were largely supported by international donors, and did not comprehensively address SRH issues including gender-based violence [31].

Gender-based violence (GBV) is common among sex workers [32]. In a systematic review of literature from Uganda, >80% of sex workers reported client-perpetrated violence while 18% experienced intimate partner violence. However, access to services after violence is often complex due to the prevailing legal context [5]. Organised engagement of law enforcement agencies and CSOs could reduce the tensions between service providers and law enforcement and enhance partnerships to improve service delivery to high risk marginalised populations including FSWs [33].

Peer involvement in facility and community-based service delivery was raised among other suggestions. These approaches could improve service uptake and client satisfaction [34, 35]. Sex workers have several formal and informal groups and leadership that can be used for demand creation and service delivery [17]. Indeed, participants mentioned that several organizations were already using these networks for service delivery. Nevertheless, these networks have a lot of challenges and limitations which would need to be addressed for their full potential to be realised.

The efforts to raise demand should be coupled with improvements in service quality including provider capacity to deliver quality services to sex workers. Training for providers should highlight policies and provider expectations that may hinder acess to services by FSWs, and the need for flexibility in implementing these policies.

## Conclusions

In conclusion, this study shows that despite the increased availability of services for FSW in Uganda, FSW continue to face major challenges in accessing these services. There is need for more comprehensive multilevel interventions that address individual, community, structural and policy barriers to access. To enhance the quality of services, improvemtns should focus on respect for clients, confidentiality, and adjustment of policies and implementation approaches that may hinder access to services including partner engament and testing, and access to treatment at multiple venues. Further, a review of distribution channels for treatment and prevention supplies, expansion of the package of interventions and organised engagement of law enforcement personnel is required.

## Abbreviations

ART: Antiretroviral therapy
ARVs: Antiretroviral drugs
CSOs: Civil society organizations
FGD: Focus group discussions
FSW: Female sex workers
GBV: Gender based violence
HIV: Human immunodeficiency syndrome
PEP: Post-exposure prophylaxis
PMTCT: Prevention of mother-to-child HIV transmission
PrEP: Pre-exposure prophylaxis
UNAIDS: Joint United Nations Program on HIV/AIDS
